# Confined Motion: Motility of Active Microparticles in Cell-Sized Lipid Vesicles

**DOI:** 10.1021/jacs.2c05232

**Published:** 2022-07-22

**Authors:** Shidong Song, Antoni Llopis-Lorente, Alexander F. Mason, Loai K. E. A. Abdelmohsen, Jan C. M. van Hest

**Affiliations:** † Department of Chemical Engineering and Chemistry, Department of Biomedical Engineering, Institute for Complex Molecular Systems (ICMS), Eindhoven University of Technology, Het Kranenveld 14, 5600 MB Eindhoven, The Netherland; ‡ Institute of Molecular Recognition and Technological Development (IDM); CIBER de Bioingeniería, Biomateriales y Nanomedicina (CIBER-BBN); Universitat Politècnica de València, Camino de Vera s/n, 46022 Valencia, Spain

## Abstract

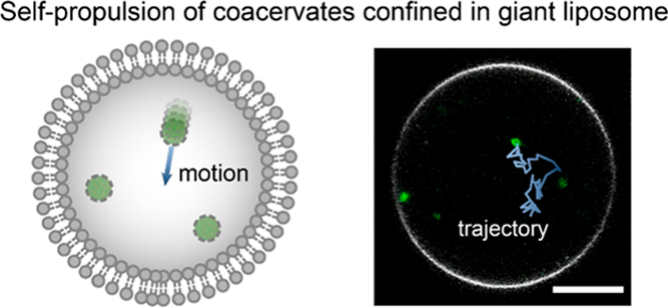

Active materials can transduce external energy into kinetic energy at the nano and micron length scales. This unique feature has sparked much research, which ranges from achieving fundamental understanding of their motility to the assessment of potential applications. Traditionally, motility is studied as a function of internal features such as particle topology, while external parameters such as energy source are assessed mainly in bulk. However, in real-life applications, confinement plays a crucial role in determining the type of motion active particles can adapt. This feature has been however surprisingly underexplored experimentally. Here, we showcase a tunable experimental platform to gain an insight into the dynamics of active particles in environments with restricted 3D topology. Particularly, we examined the autonomous motion of coacervate micromotors confined in giant unilamellar vesicles (GUVs) spanning 10–50 μm in diameter and varied parameters including fuel and micromotor concentration. We observed anomalous diffusion upon confinement, leading to decreased motility, which was more pronounced in smaller compartments. The results indicate that the theoretically predicted hydrodynamic effect dominates the motion mechanism within this platform. Our study provides a versatile approach to understand the behavior of active matter under controlled, compartmentalized conditions.

## Introduction

Nano- and micromotors are a class of materials able to harness free energy from their surroundings and transform it into kinetic energy.^1−11^ This ability, as well as their wide application window, has incited a considerable interest in active materials to further explore their adaptability, versatility, and functionality. The vast majority of research has focused on controlling the intrinsic parameters of the motor systems that govern their activity, such as the size, shape, and asymmetric placement of motile units.^12−17^ External factors that have been studied mainly concern the application of different energy sources, which can vary from chemical fuels to light- and magnetic field-induced motion.^4,11,18−20^ With these external forces, it has proven to be possible to induce life-like behavior, such as directed motion (e.g., chemo- and phototaxis)^21−24^ and swarming behavior,^25−29^ when single motile particles are able to interact with each other in a concerted fashion. In all of these cases, motile behavior is regarded as a bulk property. One aspect that has however hardly been taken into account is that in living systems, motion is often restricted by the confined space in which the motile objects operate. For example, this feature is apparent in the motility of bacteria in biofilms and the restricted motion of blood cells in capillary veins. Therefore, to improve our understanding of how motile particles move under real-life conditions, confinement should be taken into account.

Still, only a limited number of theoretical and even fewer experimental studies have investigated the motion of microparticles near 2D surfaces or under microfluidic confinement.^30−38^ In general, these studies focus on the influence of the motor architecture and composition and the topology of the environment (e.g., dimensional space and surface pattern). Different types of motion, including diffusion, sliding along the wall, and docking and unclogging, were observed experimentally for Janus-type microswimmers as a result of varying the size of confinement.^33^ For example, Liu et al.^36^ investigated a self-propulsive bimetallic swimmer in linear and curved microfluidic channels—both experimentally and with numerical simulations. They observed enhanced motion upon confinement as a result of an increased self-generated electric field, which acted as the motors’ driving force. They predicted a further increase in velocity upon decreasing the size of confinement. On the contrary, Khezri et al. reported an experimentally observed reduction in the velocity of copper/platinum bimetallic swimmers upon confinement in microfluidic channels.^37^ Moreover, decreasing the size of the channels resulted in a significant decrease in velocity. Besides bimetallic self-electrophoretic motors (which move by the generation of a local electric gradient), diffusiophoretic motors (which move by the generation of a local gradient of decomposition products) have also been studied under confinement. However, two theoretical studies reported opposite results—one predicted an increase in velocity in spherical confinement^35^ and the other predicted a slow-down when motors were near the confining boundaries.^34^ Both studies attributed the changes in velocity to the interaction between the boundaries and the chemical concentration gradients generated by the motors. It is clear from all these reports that a complex, yet poorly understood interplay exists between the confinement topology and the propulsion mechanism, which is responsible for dictating motion dynamics in confined spaces. There is thus a clear need for robust experimental systems that allow the validation of the theoretical models that have been proposed.

A probable cause for the lack of experimental data is the difficulty of establishing such a platform in which motile behavior can be effectively studied in a 3D confined space. In this paper, we report the experimental realization of compartmentalized micromotors in the interior of 3D, semipermeable micron-sized vesicles, which enables us to systematically study their motile behavior under confinement. In particular, we show the compartmentalization in giant unilamellar vesicles (GUVs) of active soft particles, composed of coacervates, surface-decorated with enzyme motile units. We demonstrate their restricted autonomous movement, when compared to unrestricted, bulk situations. By analysis of the motors’ mean-square displacement (MSD), we could identify that the motile systems attained anomalous diffusion coefficients, which meant that they show a remarkable sub-diffusive behavior in the absence or presence of relatively low concentrations of chemical fuel; normal diffusivity was restored upon increasing the substrate concentration. These effects were systematically studied as a function of GUV size, motor density, and fuel concentration. Based on these results, we can conclude that 3D confinement leads to the restricted motion of motor systems.

## Results and Discussion

**Figure 1:**
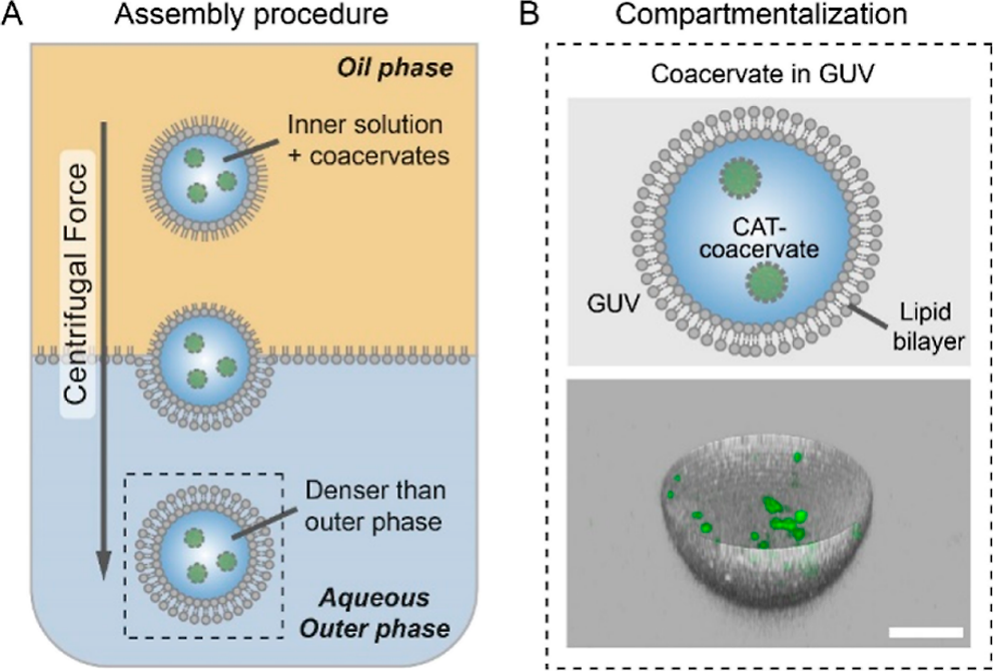
Assembly of GUVs containing active coacervate particles. (A) Schematic illustration of coacervate motors being encapsulated in GUVs via the droplet-transfer method. (B) Upper image shows a cartoon of compartmentalized CAT-coacervates inside a GUV. Lower image displays a 3D confocal image reconstituted from confocal image stacks (green: catalase which was modified with cyanine 5, gray: RhB-DOPE as a marker of the lipid membrane). Scale bar represents 10 μm.

The construction of our confined motile platform is depicted in Figure 1. As active particles, we employed our previously developed enzyme-functionalized coacervate microdroplets (diameter 1.2 ± 0.4 μm, zeta potential 1.3 ± 0.5 mV, see Figures S1 and S2).^39^ In brief, coacervates were formed by complexation of two oppositely charged amyloses (carboxymethylated and ammonium quaternized, respectively), followed by the addition of a mixture of azide-functionalized block polymer and non-functionalized terpolymer that together formed a stabilizing and fluidic membrane on the coacervate surface. Afterward, dibenzocyclooctyne-modified catalase enzymes (CAT) were attached to the coacervate membrane through a strain-promoted alkyne–azide cycloaddition reaction. Thereafter, we encapsulated the enzyme-functionalized coacervates in GUVs employing an inverted emulsion technique (also known as the droplet-transfer method) to construct our compartmentalized motor system. First, we prepared a mixture of lipids in paraffin oil, to which an aqueous phase containing the coacervates was added. Upon emulsification, lipid-stabilized water-in-oil droplets were formed, and the mixture was then layered on top of an aqueous phase for centrifugation (Figure 1A). The centrifugal force led to a transfer of droplets through the interface containing a single layer of lipid molecules, forming bilayered GUVs that sedimented at the bottom of the centrifugal tube, from where they could be harvested and purified (see the Supporting Information for details). Both the lipid GUV bilayer and the coacervate particles were labeled with complementary markers (RhB-DOPE and Cy5-catalase, respectively), which allowed their visualization by fluorescence confocal microscopy. In our system, Coulombic interactions between the coacervates and the lipid surface are not expected based on the coacervate’s near-neutral charge and the lipid surface being pegylated. Importantly, the addition of coacervate particles did not compromise the assembly and integrity of the GUVs (Figures S3 and S4), and 3D confocal imaging confirmed the successful integration of coacervates inside the lipid microcompartment (Figure 1B).

**Figure 2:**
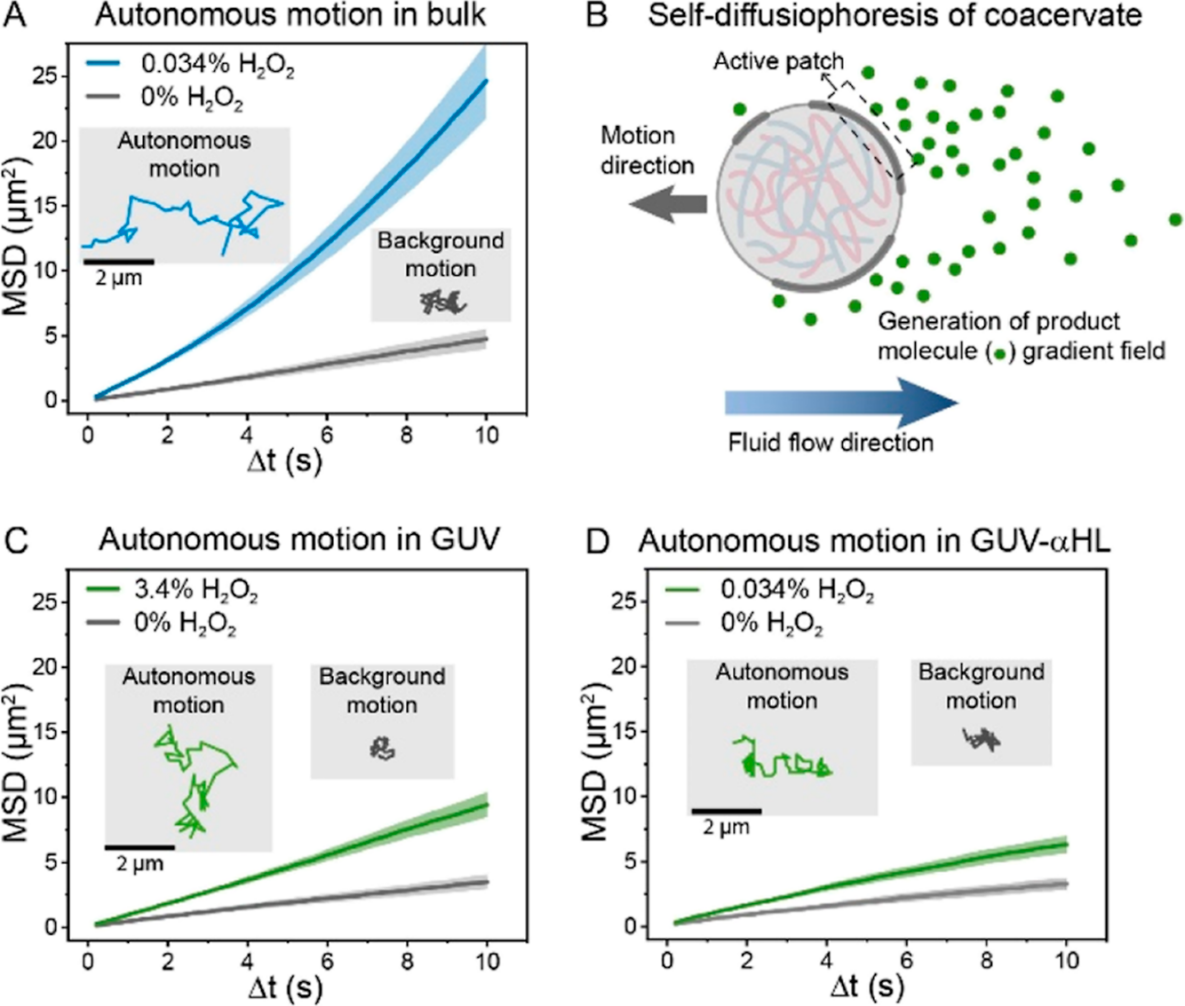
Investigation of CAT-coacervates’ motion dynamics under GUV confinement. (A) Motility profile of CAT-coacervates in bulk solution. A parabolic increase in MSD curves was observed upon addition of fuel. (B) Schematic illustration of self-diffusiophoresis of coacervates in solution. Active patches (catalase dynamic clusters) along the coacervate membrane release product molecules in the surrounding, leading to an asymmetric product molecule gradient field and therefore an osmotic imbalance. Subsequent fluid flow induced by osmotic pressure eventually results in the motion of coacervates. (C) Motility profile of CAT-coacervate motors in GUVs. A significant increase in the MSD curve and an expansion in trajectory were observed upon addition of fuel. (D) Motility profile of CAT-coacervate motors in GUVs with inserted α-hemolysin. Data of MSD curves (A,C,D) are represented as mean ± SEM.

Having confirmed the encapsulation of coacervates in GUVs, we first set out to investigate their autonomous motion under non-compartmentalizing conditions (i.e., in bulk). For this purpose, we employed the same CAT-coacervates as used for the compartmentalization approach at the same concentration (see the Supporting Information for details). Their motility was recorded in the presence or absence of their substrate, H_2_O_2_, by bright-field microscopy (five frames per second, for 60 s). Considering the diameter of the coacervate motors (∼1.2 μm), they would possess a rotational diffusion coefficient (τ_R_) ranging between 1.5 and 3 s—therefore five frames per second (0.2 s time interval) is indeed sufficient to capture the details of motile behavior of the coacervates. During tracking, *XY* trajectories were recorded, and the *Z* position was carefully adjusted so that the tracked particles were in focus during videos—movement in the *Z* axis is not considered to affect tracking along the *XY* axes. This is in line with the previous work by Sanchez et al., which demonstrated that the *XY* trajectories adequately reflect the particles’ motion in space and that the *Z* trajectories do not critically impact the analysis results.^40^ Subsequently, we analyzed the *X*, *Y* trajectories and calculated the MSD of ca. 40 coacervate particles, from multiple videos, by using a previously available tailor-made Python script (see the Supporting Information for details).^13,41−44^ In the absence of fuel, typical Brownian motion with linear MSD fitting profiles (Figure 2A) was observed for the CAT-coacervates. Upon addition of H_2_O_2_ (0.034% v/v), CAT-coacervates displayed enhanced diffusion with expanded trajectories and significantly increased MSD profiles, leaning toward a parabolic curve (Figure 2A). This behavior was in line with previous findings.^39^ Interestingly, Lyu et al. recently reported minimal propulsion of asymmetric catalase-coated silica microparticles;^45^ yet our coacervate motors differ in size, particle composition, attachment method, and their dynamic (stochastic) distribution of catalase. The motion of our catalytically active coacervates is attributed to a stochastic process, in which enzymes transiently cluster into patches that create asymmetry of the motile units on the particle surface, leading to enhanced diffusion. The mechanism of motion is considered to be self-diffusiophoretic in nature (Figure 2B); that is, by converting the fuel, the particles intrinsically create a product gradient surrounding their active patches (where catalase molecules cluster), which causes an osmotic imbalance and results in enhanced propulsion of the particles (Figure 2B). In the literature, enzymatic species have been reported to produce fluid pumping due to local density changes when directionally oriented on planar surfaces^9,46−49^—yet in coacervates, this effect is generally considered negligible (compared to self-diffusiophoresis) due to their distinct features.

Next, we studied the motility of the CAT-coacervates within GUV confinement. First, coacervates-in-GUVs were diluted in an aqueous phase (in the presence or absence of enzymatic substrate as fuel, i.e., H_2_O_2_) and subsequently transferred to an experimental chamber (see the Supporting Information for details). To record the coacervate trajectories, we examined their motion using bright-field microscopy as it allowed us to combine the observation of both the GUV compartment and the inner coacervate motors with fast image acquisition. We tracked coacervates initially located far from the boundary (i.e., located around the GUV center). In the absence of fuel, coacervates confined in GUVs underwent motion (Movie S1) with relatively short paths, which translated into a flattened MSD profile (Figure 2C). In the presence of fuel, we observed enhanced propulsion (Movie S2) as a result of catalase-mediated decomposition of H_2_O_2_ into H_2_O and O_2_, which translated into expanded trajectories and a significant increase in the MSD profile (Figure 2C). Remarkably, comparing the MSD profiles of non-compartmentalized versus compartmentalized coacervates (Figure 2A,C), we found striking differences in their autonomous motion behaviors: not only did the compartmentalized coacervates exhibit lower MSD values in the absence and presence of fuel but noteworthily the shape of the MSD curves (in the presence of fuel) changed from concave upward for the non-compartmentalized particles to nearly linear for the compartmentalized ones (indicative of restricted motion). The permeation of H_2_O_2_ through the lipid bilayer has previously been measured to be fast, in the order of milliseconds (permeability coefficient = 1.1 × 10^–6^ m s^–1^, lipid bilayer thickness ≈ 3.7 nm).^50^ Yet, to further confirm the effect of confinement on the motion dynamics and rule out the effects of membrane-induced limited diffusion of substrate/products, we monitored the coacervates’ motion in GUVs comprising a highly permeable membrane (i.e., with the inserted α-hemolysin membrane pores, see the Supporting Information for details) at the same H_2_O_2_ concentration as in bulk. The MSDs resulting from coacervate motion upon addition of H_2_O_2_ (0.034% v/v) (Figure 2D) showed a similar MSD curve shape as displayed in Figure 2C, thus indicating that the anomalous motion dynamics were not affected by substrate diffusion issues but a result of the confinement effect.

The change in motion dynamics under confinement, when compared to unrestrained conditions, allowed us to probe different theories that describe confined motion.^34,35^ For this purpose, we systematically investigated three parameters which we expected to have an effect on motility, namely, fuel concentration, GUV confinement size, and coacervate motor density.

**Figure 3:**
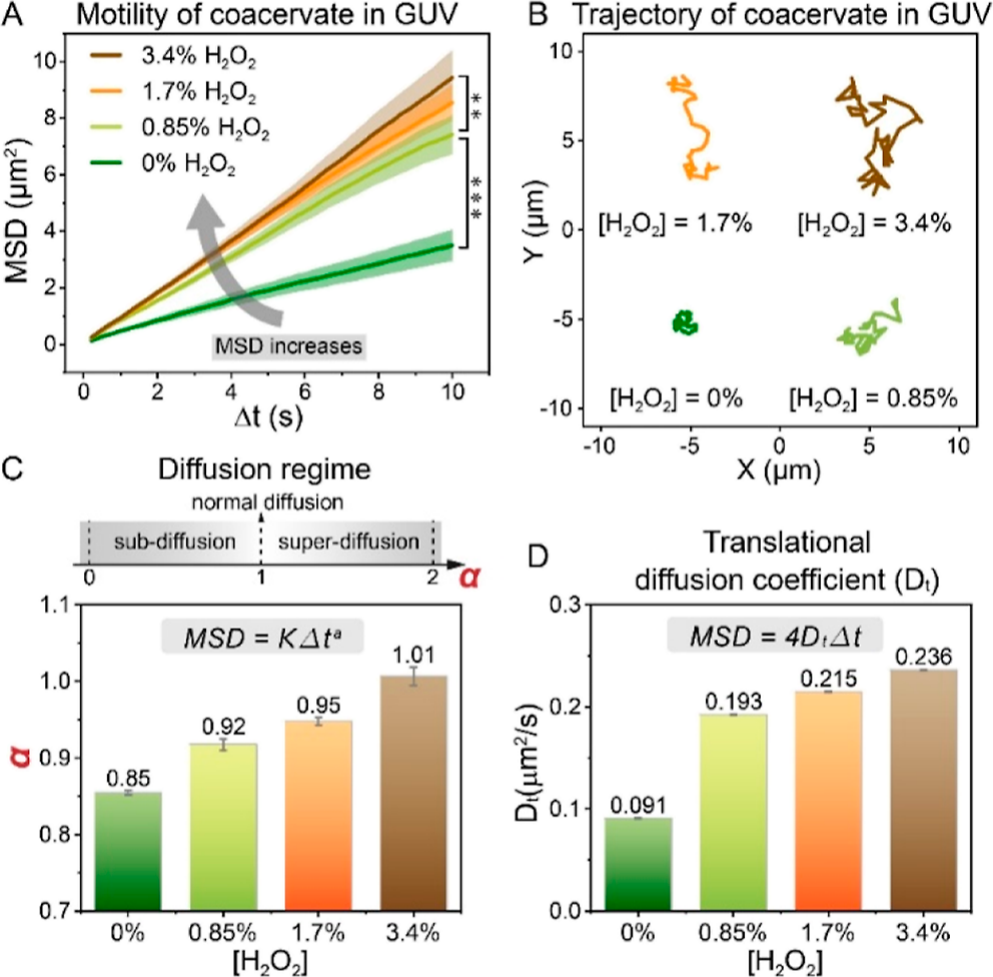
Investigation of motion dynamics depending on fuel concentration. (A,B) MSD profiles and trajectories of CAT-coacervates in GUVs with different hydrogen peroxide concentrations. Higher fuel concentration resulted in an increased MSD and expanded trajectory. MSD curves are significantly different from each other as indicated. *P* values were calculated using a *t*-test (two-tailed). **P < 0.01 and ***P < 0.001. (C) Diffusion regime indicated by the anomalous exponent α. With [H_2_O_2_] rising from 0 to 3.4%, α increased from 0.85 to 1.01, suggesting a transition from sub-diffusion to normal diffusion. (D) Translational diffusion coefficient values at different fuel concentrations.

Having confirmed that the GUV confinement altered motion regimes, we set out to investigate the extent of the confinement effect on motion dynamics by studying the crossover of different motion regimes in confinement. We therefore performed motility experiments of coacervates-in-GUVs at different fuel concentrations. A direct relationship between the MSD values and fuel concentrations was observed (Figure 3A), with higher H_2_O_2_ concentration leading to faster motion and more expanded trajectories (Figure 3B). Interestingly, in the presence of H_2_O_2_, the MSD curves deviated from a straight line at higher time intervals (Δ*t* > 6 s), resulting in a concave downward shape (see Figure S5). Generally, when colloidal particles undergo Brownian motion, that is, normal diffusion, they exhibit an MSD that is linear in time—a deviation from such trend is indicative of anomalous diffusion. Mathematically, anomalous diffusion is described as MSD = *K*Δ*t*^α^.^51^ Here, α is the anomalous exponent that indicates how far the motion deviates from normal Brownian motion: (i) α = 1 indicates the Brownian motion, (ii) α > 1 is indicative of a superdiffusive process, and (iii) α < 1 is indicative of a sub-diffusive process. To gain further insights into the motile behavior of the confined coacervates, MSD curves were fitted with MSD = *K*Δ*t*^α^ to obtain the anomalous exponent α (Figure S6). The resulting α values at different fuel concentrations are summarized in Figure 3C. Noteworthily, there is a clear trend toward lower α at lower fuel concentration. In the absence of fuel, the α value for coacervates-in-GUVs was 0.85, indicating constrained motion (sub-diffusion); in comparison, fitting of MSD plots of coacervates in bulk solution, in the absence of fuel (Figure 2A), resulted in an α of 1.00 (normal diffusion). This further confirmed that the motion behavior was altered by confinement imposed by the GUV membrane. When the hydrogen peroxide concentration increased, the α values steadily increased and reached 1.01 at 3.4% v/v H_2_O_2_ (Figure 3C). Such increase in α indicated a transition from sub-diffusion toward normal diffusion upon addition of fuel, which means that the autonomous motion of the particles was able to compensate for the confinement effect. In addition, the translational diffusion coefficient (*D*_T_) was obtained by fitting MSD profiles with the equation MSD = 4*D*_T_ Δ*t* (assuming α = 1, based on the fact that the effect of confinement is almost negligible at Δ*t* < 6 s—and only becomes dominant at Δ*t* > 6 s). By applying this formula, we obtained a 2-fold increase in *D*_T_ when the H_2_O_2_ concentration increased from 0 to 0.85% (Figure 3D). A further increase in *D*_T_ at higher fuel concentration reflects the enhanced self-propulsion of coacervates under confinement. We also applied the equation MSD = 4*D*_T_ Δ*t* to the first 3 s of the MSD curves and derived *D*_t_ values (Figure S7), as at this time regime, there is no effect of confinement (which can also be seen in the linear curve profile). Indeed, the resulting *D*_t_ are almost similar to those shown in Figure 3D, suggesting that assuming that α for all cases is 1 does not change the overall increasing trend of *D*_t_. Altogether, these results indicated an enhanced self-propulsion and crossover from sub-diffusive toward normal diffusion of active particles in GUV confinement when increasing the fuel concentration.

**Figure 4:**
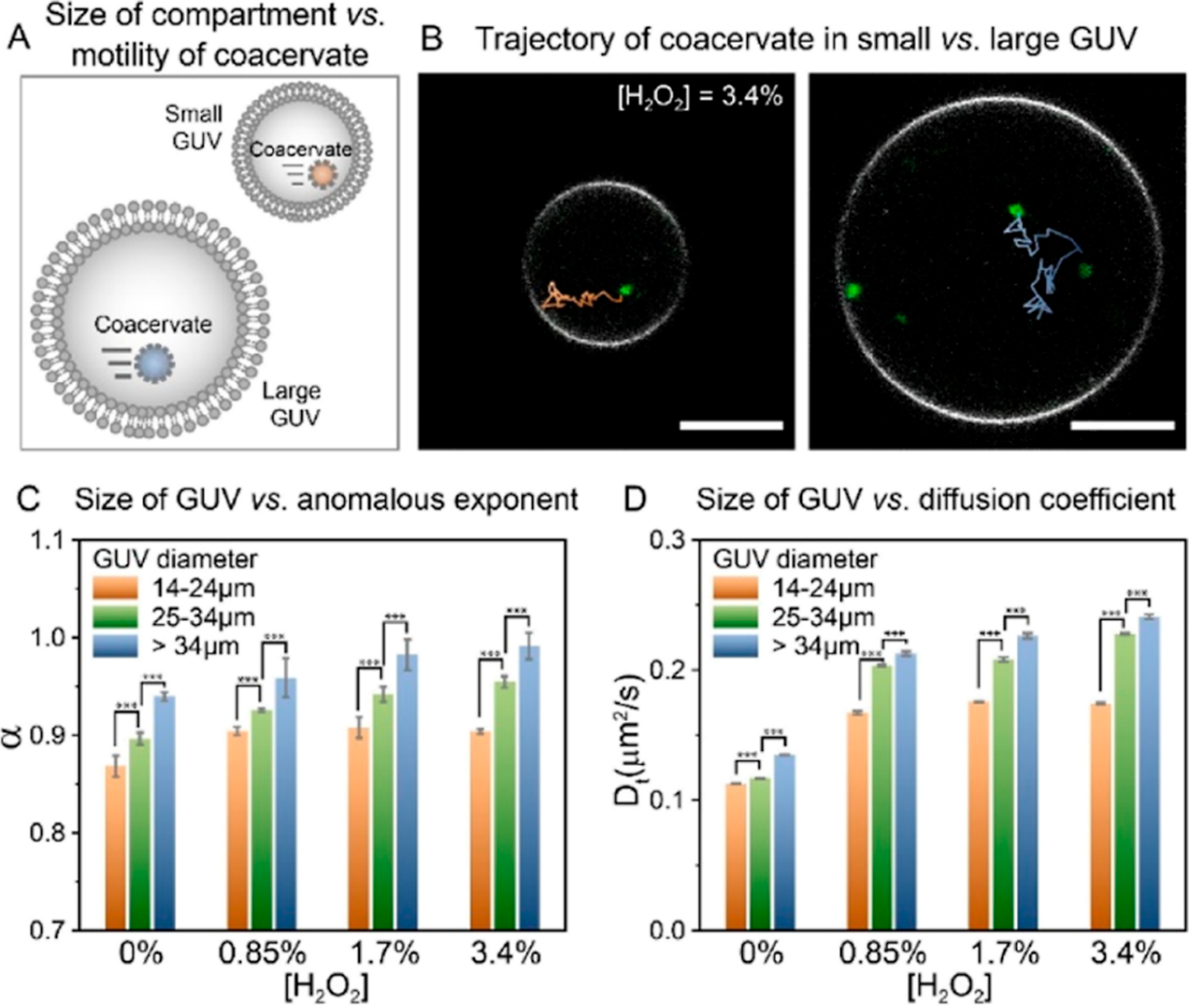
Investigation of motion dynamics depending on the compartment size. (A) Schematic illustration of coacervates in small and large GUVs. (B) Trajectories of coacervates in small and large GUVs with [H_2_O_2_] of 3.4%. Bright-field tracking is overlaid on top of confocal images of coacervates in GUVs. Scale bars represent 10 μm. (C,D) Anomalous exponent and diffusion coefficient depending on the GUV size, at different fuel concentrations. For each size group, 10–20 coacervates were analyzed at each [H_2_O_2_]. Data in C and D are represented as mean ± SD. Statistical analysis was performed to assess statistically significant differences between pairs of data as indicated. *P* values were calculated using a *t*-test (two-tailed). ***P < 0.001.

Subsequently, we investigated the correlation between compartment size (i.e., GUV diameter) and motility of the confined active particles. Our hypothesis was that smaller GUVs would display a more pronounced confinement effect as the hydrodynamic interaction between coacervates and the membrane would be increased. Previous research^34^ reported that the viscous dragging (σ) is inversely affected by the distance to the boundary (*L*) (σ = μ*u*_s_/*L*, where μ is the viscosity of the medium and *u*_s_ is the phoretic slip). GUVs assembled by the droplet-transfer method show a relatively wide size distribution (Figure S3)—thus, we set to analyze coacervate motion in GUVs categorized in three different diameter groups: small (14–24 μm), medium (25–34 μm), and large GUVs (>34 μm). In our case, this distance (*L*) can be considered approximately as the radius of the GUV compartment as we tracked coacervate particles that were initially close to the GUV center. We compared trajectories, the anomalous diffusion exponent (α), and diffusion coefficient (*D*_T_) of the different size groups (Figure 4) at different fuel concentrations. Indeed, as depicted in representative trajectories in Figure 4B, coacervates in small GUVs were more prone to be in close proximity of the GUV membrane in their path, whereas coacervates in larger GUVs had more space to diffuse around, being less restrained in their movement. This observation correlated with the extracted α and *D*_T_ values (Figure 4C,D). At all fuel concentrations, a positive correlation between α and GUV size was observed, with a sub-diffusion regime in smaller GUVs and a normal diffusion in larger GUVs at 3.4% v/v H_2_O_2_. This progressive increase in α values upon increasing the GUV size suggests a proportional decrease of viscous dragging with distance in line with ref (34). A similar trend was observed for *D*_T_, indicating a more effective propulsion of coacervates in larger GUVs. Noteworthily, for the small size group (14–24 μm), α and *D*_T_ increased when the concentration of H_2_O_2_ increased from 0 to 0.85% v/v; a further increase in fuel concentration did not alter both values. In contrast, for medium (25–34 μm) and large GUV (>34 μm) sizes, we observed a continuous increase in α and *D*_T_ from 0 to 3.4% v/v H_2_O_2_. Thus, under the investigated conditions, small GUVs (14–24 μm) severely restrict the motility of coacervates to the point that enhanced motion reached its limit and additional chemical energy could not be converted into additional motion. Interestingly, this may suggest an interplay between enhanced motion, distance to the boundary/viscous dragging, and the physical boundary itself: in small GUVs, coacervates are more likely to get relatively closer to the boundary as they move with their (enhanced) Brownian motion (thus, more fuel increasing this probability), but at the same time, as they move closer, this correlates with an effective increase in viscous dragging—thus, upon increasing the fuel concentration in small GUVs, the enhanced coacervate motion and viscous dragging counteracted each other. In addition, regarding the hydrodynamic/viscous dragging effect, it is worth considering that the ratios between the GUV compartments and the coacervates are ∼11 and ∼30 for the small and large GUVs, respectively, and in line with our observations, previous reports found motility severely restricted in the 1.5–5 confinement/particle size ratio range, yet their model/calculation was not extended to larger ratios—thus, the ratio confinement/particle size is an important parameter to consider in the development of models that advance the understanding of motility under confinement. Altogether, our results indicate that a decrease in GUV compartment size correlates with a decrease in the motility of active particles.

**Figure 5:**
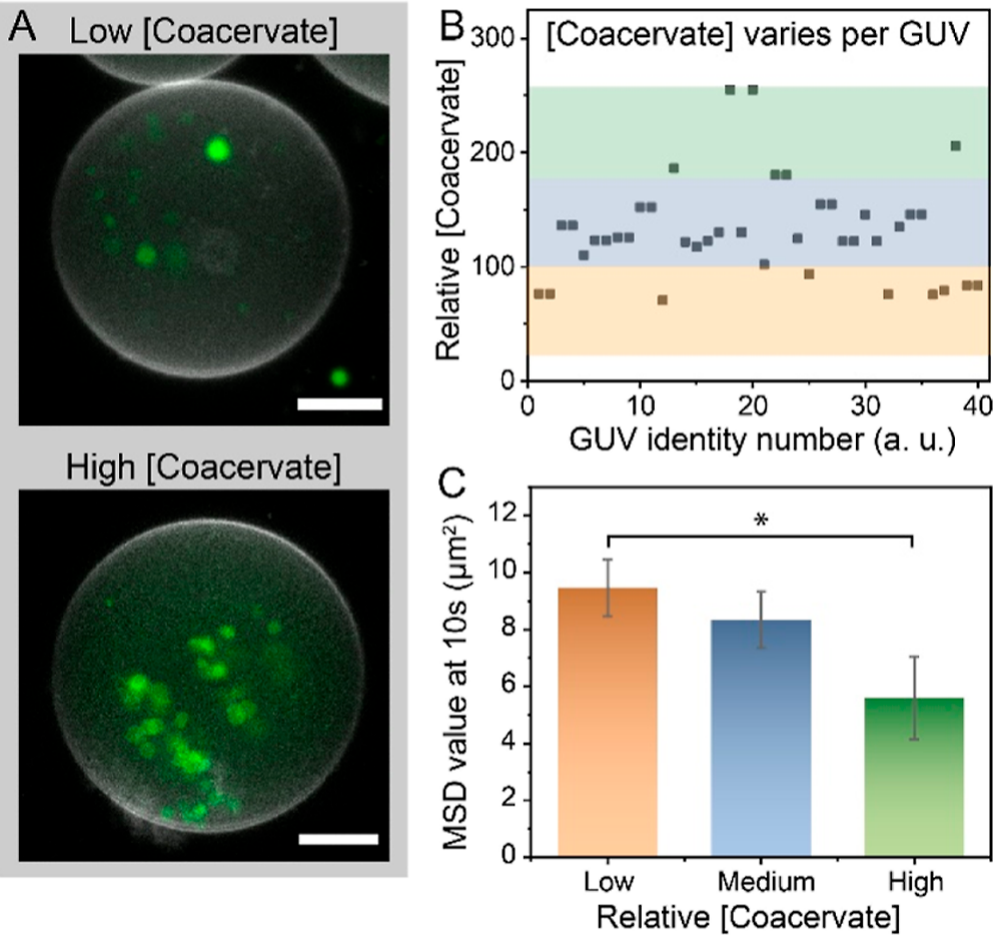
Investigation of coacervate concentration and its effect on motion dynamics. (A) Projection of confocal image stacks of two representative GUVs with high and low coacervate concentrations. The scale bar represents 10 μm. (B) Relative [coacervate] for different GUVS. 40 GUVs were analyzed using confocal image stacking to determine the relative [coacervate]. The coacervate concentrations were (arbitrarily) grouped in three regions: the [coacervate] lower than 100 a.u. was denoted as low, the [coacervate] between 100 and 175 a.u. was denoted as medium, and the [coacervate] higher than 175 a.u. was denoted as high. (C) With 0.85% hydrogen peroxide, the MSD values of coacervate motors in a time frame of 10 s were categorized into the three [coacervate] groups: low, medium, and high. Statistical significance between conditions is indicated by the asterisk (*p < 0.05).

Finally, we set out to investigate if differences in the relative concentration of coacervates per compartment could influence their motile properties. We hypothesized that the behavior of active particles could be affected by the presence of other active particles in their surroundings due to, for example, potential proximal particle–particle interactions. It would be of interest to experimentally validate this hypothesis to know if the concentration of active particles should be considered in future theoretical models. Therefore, we carried out experiments acquiring z-stacks of coacervates in GUVs (by confocal microscopy, Figure 5A) prior to recording the motion of the compartmentalized particles (by bright-field microscopy, upon addition of 0.85% H_2_O_2_ as fuel). In all cases, 45 images along the *z*-axis were captured, revealing fluorescent signals of coacervates located at different planes. Then, the total fluorescence (sum of the 45 planes) of each GUV (*N* = 40) was used as an estimation of their relative coacervate concentration (see the Supporting Information and Figure S8 for details). As depicted in Figure 5B, most GUVs had a relative coacervate concentration within a range of 100–175 arbitrary units (a.u), yet we observed some heterogeneity with a fraction of GUVs located above (>175 a.u., high concentration) and below this range (<100 a.u., low concentration). Interestingly, when we extracted the motion of coacervates for each group, we found larger MSD values for confined coacervates of the low coacervate concentration group (at 10 s, MSD = 9.5 ± 1.0 μm^2^), as compared to the medium (at 10 s, 8.3 ± 1.0 μm^2^) and high concentration group (at 10 s, 5.6 ± 1.5 μm^2^; significant difference with *p* < 0.05 compared to the low conc. group) (Figure 5C). The corresponding MSD plots are shown in Figure S9, which demonstrate non-ballistic behavior regardless of the coacervate concentration. A similar trend was determined when narrowing the GUV diameter range down to 25–34 μm (Figure S10). Previous research by Donado et al. also found restricted motility at high particle concentrations in the case of magnetically powered (1 mm) particles on a planar surface.^52^ In order to clarify the influence of particle concentrations on the intrinsic Brownian motions, we carried out experiments in the absence of H_2_O_2_ fuel. No differences were observed between the three concentration groups in the absence of fuel (Figure S11). Accordingly, these data suggest that the active particle motion in confinement is affected by the overall particle concentration, in a way that relatively high concentrations result in decreased motion which could be attributed to a distortion of the product gradient field by the surrounding particles and by a local faster fuel consumption.

Our results reveal several interesting effects about the behavior of active particles within the 3D confinement. First, the motion of such compartmentalized particles is decreased as compared to the same particles free in solution. Furthermore, compartmentalized particles move in a sub-diffusion regime and the presence of fuel compensates for the confinement effect and enhances motility toward normal diffusion. In contrast, the same non-compartmentalized particles move under Brownian motion in the absence and with (superdiffusion) ballistic motion in the presence of fuel. Third, the size of the compartment matters, with a stronger sub-diffusive effect (i.e., restricted motion) in smaller compartments. Finally, particles are influenced by the relative concentration of peers in their surroundings, with higher concentrations leading to a decreased motion.

It has been theoretically proposed^34,35^ that there are two competing phenomena that influence the motion of swimmer particles in confinement: (i) the hydrodynamic effect: the velocity of fluid flow diminishes to zero toward the confinement wall (the so-called “no-slip condition”, Figure S12A), which results in particles experiencing a viscous fluid drag and a diminished motion, and (ii) the phoretic effect: the confinement boundary affects the transport of product molecules, as there is less space for the product molecules to diffuse, leading to a more pronounced product gradient around the particle as depicted in Figure S12B (higher local concentration of product asymmetrically distributed around the particle), resulting in increased motion. As mentioned above, our catalytically active particles move by self-diffusiophoresis, and the product molecule gradient is the key driving factor for self-propulsion. Among the two competing effects (hydrodynamic effect and phoretic effect), our findings suggest that the decelerating hydrodynamic effect is the dominating one for our chemically fueled swimmers, as we see an overall decrease in motion upon confinement. In fact, when there is no product gradient (i.e., in the absence of fuel and therefore the phoretic effect can be ruled out), the difference in motion of non-compartmentalized (Brownian) versus compartmentalized particles (sub-diffusive) confirms that the hydrodynamic effect influences motion. Addition of fuel leads to the enhanced propulsion of the particles, which partially counteracts the hydrodynamic effect, yet motion is still partially restricted as compared to their non-compartmentalized counterparts. Additionally, our results also indicate that active particles in smaller compartments experience a larger hydrodynamic drag (i.e., more restricted motion). These findings are in agreement with other reports which describe a deceleration of particles near solid surfaces and in microfluidic channels.^31,34^ In contrast, Popescu and co-workers theoretically predicted an increase in velocity for a diffusiophoretic motor in a spherical (impermeable) compartment,^35^ caused by the phoretic effect and despite the opposing hydrodynamic drag caused by the confinement boundaries. However, only impermeable confining walls were considered in their study, in which case the phoretic effect is more pronounced (by preventing the equilibration of the product gradient). Our phospholipid vesicles are highly permeable to oxygen, and therefore, the phoretic effect is expected to be mitigated in our system (Figure S12). The contradiction between their theory and our experimental results can therefore be explained by the semipermeability of the GUV membrane.

## Conclusions

In conclusion, we have presented a study about the confined motion dynamics of catalytically active particles in cell-sized lipid vesicles. The fabrication methodology, based on the direct encapsulation of pre-formed particles during GUV formation, is versatile and could be extended to the study of other active particles (e.g., light-propelled motors). We observed that the confinement hinders the motion of coacervates and results in a sub-diffusion regime. Interestingly, addition of chemical fuel changes the behavior of coacervates inside GUVs toward normal diffusion and counteracts the confinement effect. Furthermore, we determined that the confinement effect correlates with the compartment size, with more restricted motion in smaller compartments. Finally, we observed that the overall internal coacervate concentration influences motion dynamics. These results all are in line with theoretical models that predict a leading role for the hydrodynamic effect in confined motion. Our study highlights the importance of considering the dynamics of active matter in confinement, such as in cell-like compartments, and provides a versatile platform to assess experimentally this feature of motile systems.

## Supporting Information Available

Materials and methods, Figures S1 to S12, supplemental references (PDF)
Restrained motion of coacervates in GUVs in the absence of fuel (AVI)
Self-propulsion of coacervates in GUVs in the presence of fuel. [H_2_O_2_] = 3.4% (AVI)

## Supplementary Material

ja2c05232_si_001.pdf

ja2c05232_si_002.avi

ja2c05232_si_003.avi
