# Supplementary material for: Confined Motion: Motility of Active Microparticles in Cell-Sized Lipid Vesicles
Source: J Am Chem Soc. 2022 Jul 22;144(30):13831–8. doi: 10.1021/jacs.2c05232 (PMC9354240; doi:10.1021/jacs.2c05232)
Supplement: Supplementary file 1 — ja2c05232_si_001.pdf [file ja2c05232_si_001.pdf]

Supplementary Information for

Confined Motion: Motility of Active Microparticles in Cell-Sized Lipid Vesicles

Shidong Song,† Antoni Llopis-Lorente,† Alexander F. Mason, Loai K. E. A. Abdelmohsen* and Jan C. M. van Hest*

*Loai K. E. A. Abdelmohsen. Email: l.k.e.a.abdelmohsen@tue.nl

*Jan C. M. van Hest. Email: j.c.m.v.hest@tue.nl

This PDF file includes:

Materials and methods
Figures S1 to S6
Legends for Movies S1 to S2
SI References

Other supplementary materials for this manuscript include the following:

Movies S1 to S2

Materials and Methods

Materials

All materials were used as received unless otherwise stated. Catalase (from bovine liver), sulfo-dibenzocyclooctyne-NHS ester (DBCO-NHS) and 30% hydrogen peroxide solution were obtained from Sigma-Aldrich. Hydrogen peroxide solutions used for motility experiments were prepared by sequential dilutions of 30% hydrogen peroxide solution. Sulfo-Cyanine5 NHS ester was obtained from Lumiprobe. Lipids for GUV assembly: 1,2-dioleoyl-*sn*-glycero-3-phosphocholine (DOPC), 1-palmitoyl-2-oleoyl-glycero-3-phosphocholine (POPC), 1,2-distearoyl-*sn*-glycero-3-phosphoethanolamine-*N*-[biotinyl(polyethylene glycol)-2000] (DSPE-PEG), and 1,2-dioleoyl-*sn*-glycero-3-phosphoethanolamine-N-(lissamine rhodamine B sulfonyl) (ammonium salt) (DOPE-RhB) were provided by Avanti Polar Lipids. Paraffin oil (0.86 g/cm^3 at 20 °C) was from JT Baker. Cholesterol, glucose, sucrose, bovine serum albumin (BSA), and alpha-hemolysin (αHL) from *Staphylococcus aureus* were purchased from Sigma-Aldrich.

For polymer synthesis: monomethoxy poly(ethylene glycol) (1 and 2 kDa) and azido-PEG-OH macroinitiator (3 kDa) were purchased from Rapp Polymere, trimethylene carbonate was purchased from TCI Europe. For the preparation of modified amylose derivatives, amylose (12-16 kDa) was supplied by Carbosynth and 3-chloro-2-hydroxypropyltrimethyl ammonium chloride (65 wt% in water) was supplied by TCI Europe. Modified amyloses and polymers were synthesized as previously published.[1]

Catalase modification: synthesis of CAT(Cy5-DBCO-Catalase)

Catalase was modified with Cy5 and DBCO as follows: 50 mg (0.2 µmol) catalase was dissolved in 10 mL 0.1 M sodium bicarbonate buffer (adjusted to pH = 8.2) in a glass vial, to which 1.24 mg (1.6 µmol) sulfo-Cy5-NHS ester and 0.85 mg (1.6 µmol) sulfo-dibenzocyclooctyne-NHS ester were added. This 8:1 (dye: protein) stoichiometry was chosen to ensure sufficient dye labeling as the NHS ester is easily hydrolyzed. The reaction mixture was covered with aluminum foil and stirred overnight at 4°C and then dialyzed for 48 hours against 1× PBS buffer. Cy5 labeled catalase was purified via fast protein liquid chromatography (FPLC, BioRad NGC system). The protein concentration was determined to be 4.4 mg/mL by Nanodrop. Cy5 labelling number per catalase tetramer was determined to be 0.9 and DBCO (Absorption peak at 310 nm) labelling number per catalase tetramer was determined to be 1.0 by Nanodrop.

The activity of mCAT was determined by measuring the decomposition of hydrogen peroxide over time via UV/Vis spectroscopy (JASCO V-650). In these assays, 0.1 mL of diluted catalase (unmodified or modified) in PBS solution was mixed with 2.9 mL hydrogen peroxide solution (0.036 wt% in water), and the characteristic absorbance at 240 nm (A240) was monitored over time. Enzyme activity was calculated according to:

$$\frac{\text{Units}}{\text{mL}}\text{enzyme} = \frac{3.45 \times df}{time \times 0.1},$$

where 3.45 corresponds to the decomposition of 3.45 µmoles of hydrogen peroxide in a 3.0 mL reaction mixture producing a decrease in the A240 from 0.45 to 0.40, “df” is the dilution factor and “time” is the time in minutes for A240 to decrease from 0.45 to 0.40. Enzyme activities of unmodified catalase and modified catalase were determined after 3 individual measurements to be about 10×10^3 U/mg and 9×10^3 U/mg respectively. Unit (U) definition: One unit of catalase decomposes 1.0 µmole of H_2O_2 per minute at pH 7.0 at 25 °C, while the H_2O_2 concentration decreases from 10.3 mM to 9.2 mM.

Fabrication of CAT functionalized coacervates

Coacervates were prepared based on a sonication method as previously reported:[1] to 200 μL 0.5 mg/mL quaternized amylose (Q-Am) solution in an Eppendorf tube was added 100 μL 0.5 mg/mL carboxymethyl amylose (Cm-Am) solution to induce coacervation. The mixture was placed in a sonication bath for 2 minutes to allow coacervate growth, followed by addition of 10 μL 50 mg/mL polymer mixture (20%v/v 50 mg/mL azido-PEG_{68}-*b*-PCL_{50}-*g*-$PTMC_{50}$ and 80%v/v 50 mg/mL terpolymer in PEG 350) to generate a polymeric membrane and stabilize the coacervate core. The size of such coacervates was effectively limited to 1-2 μm by utilizing sonication during coacervate droplet growth.

After the assembly of coacervates, 46 μg CAT (Cy5-DBCO-Catalase) was added to allow CAT attachment to the polymeric membrane through the strain-promoted azide–alkyne cycloaddition (SPAAC) between the azido-group installed on the polymer membrane and the DBCO moiety introduced in the enzyme. The reaction was carried out for two hours, followed by separation of unbound enzymes by centrifugation and refreshing the supernatant with 1× PBS.

Preparation of giant unilamellar vesicles (GUVs) with encapsulated coacervates

Our protocol for the preparation of GUVs is based on the droplet transfer method.[2] Lipid stock solutions were prepared in chloroform and stored at -20 °C until use. Lipid solution aliquots were taken and mixed with 200 μL of paraffin oil to obtain a lipid mixture containing DOPC, POPC and cholesterol in a molar ratio of 35/35/30 (10 mM total concentration). In addition, 1% of DSPE-PEG (to prevent GUV aggregation) and 0.06% of DOPE-RhB (for membrane labelling) were also incorporated. Firstly, the lipid mixture in paraffin was heated at 80 °C for 30 min and cooled on ice for 10 min. Next, 20 μL of inner phase solution (PBS 0.8X containing the as-made coacervates, 200 mM sucrose, pH 7.4) was emulsified in the 200 μL of lipid solution by strong vortexing for 25 s while turning the reaction tube to prevent sedimentation of the water droplet. Thereafter, the emulsion was incubated on ice for 10 min. Subsequently, the mixture was layered on top of 150 μL of pre-cooled outer phase (PBS 0.8X, 200 mM glucose, pH 7.4) solution in a 1.5 mL plastic tube and immediately centrifuged at 4 °C for 20 min at 3300 rcf. GUVs were harvested by puncturing the tube at the position of the GUV pellet and dripping the aqueous phase. To remove any non-encapsulated material, GUVs were pelleted by centrifugation at 1500 rcf for 2 min after which the supernatant was replaced with 40 μL fresh outer phase, which was repeated twice.

For alpha-hemolysin insertion, GUVs were incubated with 10 μg mL^{-1} of alpha-hemolysin for at least 15 min prior to motility studies.

Confocal imaging of coacervates in GUVs

Confocal laser scanning microscopy (Leica TCS SP8) was used to capture images of catalase-coacervates in GUVs with a 638 nm laser line (for Cy5) and a 552 nm laser line (for DOPE-RhB) using a 63×, 1.20 NA water immersion objective. The pinhole was set to 1 Airy Unit (156 μm). For confocal z stacks, 45 images per GUV per laser line were taken to reconstitute 3D images.

For the analysis of the coacervate concentration, a circular ROI area (same size as the GUV) was selected, and grey value measurement (for fluorescence intensity) was performed for that ROI area of one confocal z stack (45 images). The sum of grey values from 45 images was considered as relative coacervate concentration.

For coacervate size analysis, succinylated Atto 488 modified bovine serum albumin (BSA) was loaded in the interior of the coacervates for better visualization by confocal microscopy. Images of BSA-loaded coacervates were captured with a 488nm laser line using a 63×, 1.20 NA water immersion objective. The images were then converted to binary images, and the area of each

coacervate was analyzed using software ImageJ. The diameter of coacervates was obtained by averaging at least 100 coacervates. Zeta (ζ) potential measurement was performed on a Malvern instrument Zetasizer (model Nano ZSP). Coacervates were suspended in 20 times diluted PBS during measurements. Zetasizer software was used to process and analyze the data.

Motility experiments.

Experimental chamber:

A simple experimental chamber was designed and prepared to minimize side effects that could be mistaken as self-propulsion, such as drift or solution evaporation. This chamber was made from two glass microscopy slides spaced by two pieces of autoclave tape. Autoclave tape was first attached to a larger glass slide on two ends, followed by the addition of sample in the middle, and capping by a smaller glass slide.

Optical recording:

The videos of coacervate motion were recorded using a bright-field camera (DFC7000T) on a confocal laser scanning microscope (Leica TCS SP8). A 63× water immersion objective was used for this recording. The coacervates in GUV suspension and the enzyme substrate (hydrogen peroxide) were first mixed thoroughly and immediately added to the chamber. The recording (5 fps, > 60 s per recording) started shortly after. The highest hydrogen peroxide concentration after mixing was set to 3.4%. This concentration was chosen to avoid oxygen bubble generation that could disturb the liquid inside the chamber and mask self-propulsion. The coacervate concentrations under compartmentalized and non-compartmentalized conditions were kept similar. For non-compartmentalized conditions, coacervates were diluted 10 times from coacervate stock suspension; for compartmentalized conditions, 2x concentrated coacervates were encapsulated inside GUVs, followed by 20 times dilution of GUV samples for motility experiments. The sample in the experimental chamber was replaced at least every 5 min to maintain the same substrate concentration.

Data analysis of motion: A tailor-made Python script was used to track the coacervates and obtain the mean square displacement (MSD).[3,4] MSD is a measure of deviation of the position of a particle with respect to its initial position over time. It is commonly used to analyze the dynamics of self-propelled particles and is calculated as below in 2D projection:

$$MSD\ (t) = \left\langle (\overrightarrow{x}\,(t) - \vec{x}\ (0))^2 \right\rangle,$$

where $\vec{x}(0)$ is the initial position of the coacervate, and $\vec{x}(t)$ is the position of the coacervate when time is *t*. Then the obtained MSD was plotted against the time interval Δt.

To obtain the anomalous exponent α and translational diffusion coefficient D_T, MSD curves were fitted with an exponential model MSD = $K\Delta t^\alpha$ and a linear model MSD = $4D_T\Delta t$, respectively. Both exponential model and linear model fit the experimental data well with R^2 higher than 0.99 (Figure S6).

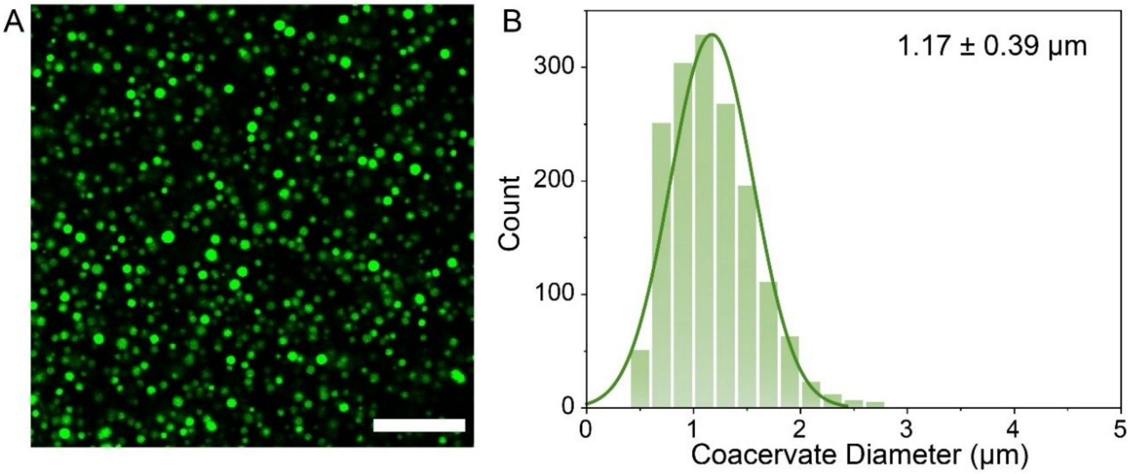

Figure S1. Coacervate size distribution. A) BSA (labelled with dye Atto488) loaded coacervates. Scale bar represents 15 µm. B) Histogram analysis of confocal microscopy images of coacervates showing an average coacervate diameter of 1.17 ± 0.39 µm.

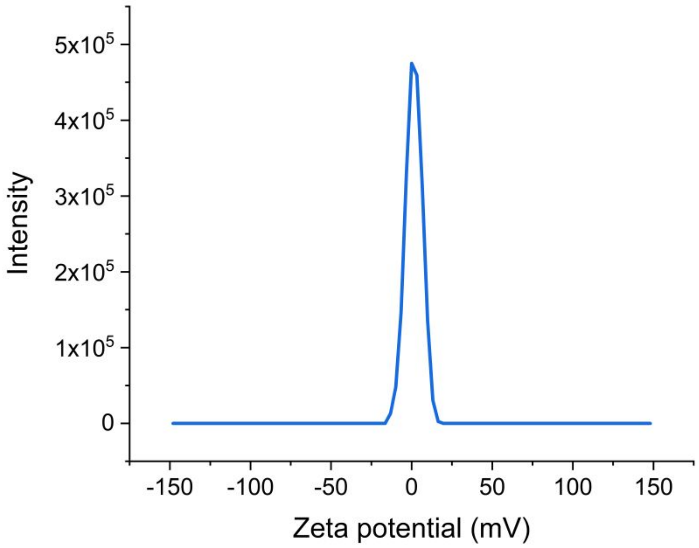

Figure S2. Zeta potential distribution of coacervate particles.

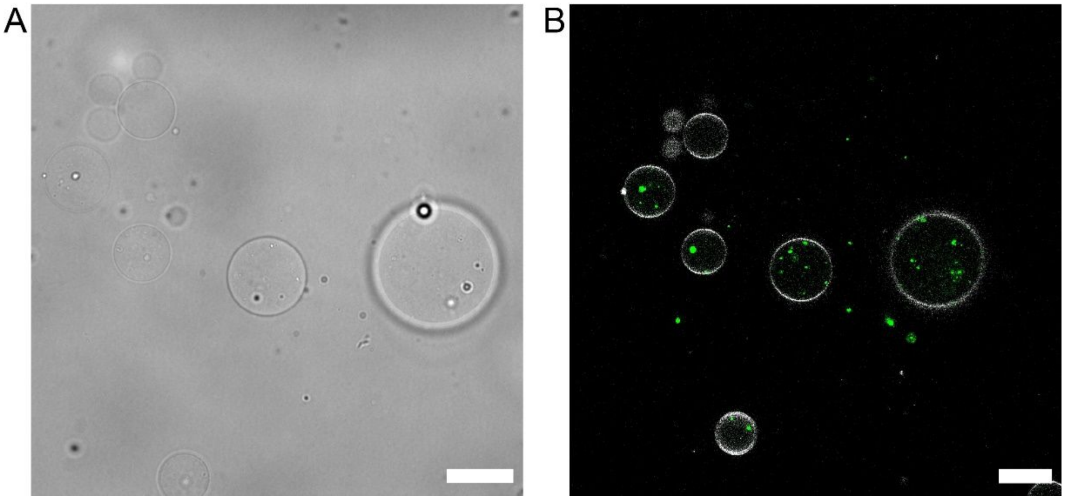

Figure S3. CAT-coacervates were successfully encapsulated in GUVs without compromising GUV integrity. A) Bright-field image of coacervates-in-GUVs. B) Confocal image (Green: catalase which was modified with Cyanine 5, grey: RhB-DOPC as marker of the lipid membrane) of coacervates-in-GUVs. Scale bar represents 20 µm.

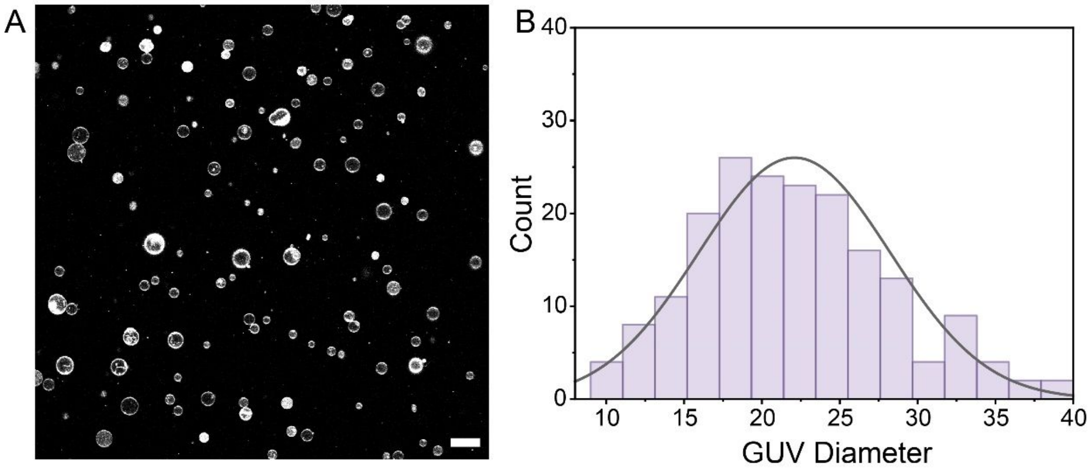

Figure S4. GUV size distribution. A) Confocal image of GUVs (with RhB-DOPC as marker) (A). Scale bar represents 50 µm. B) Histogram analysis of confocal microscopy images of GUVs showing an average GUV diameter of 22.1 ± 6.2 µm.

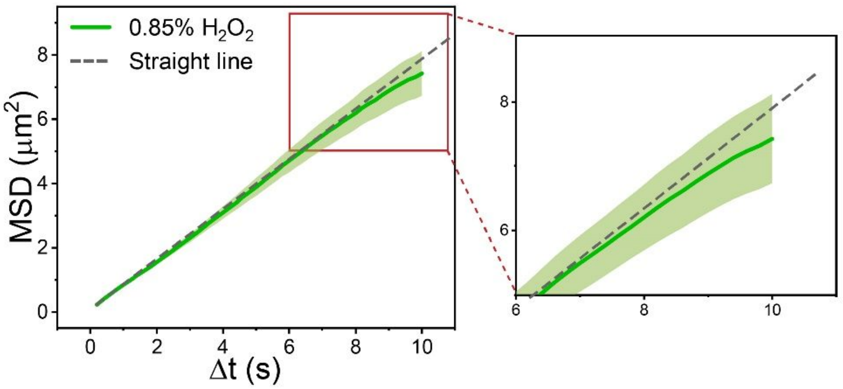

Figure S5. Comparative MSD profile of coacervates in GUVs (0.85% H_2O_2) *vs.* a straight line, and corresponding zoomed in region. It can be observed that MSD curves deviated from a straight line at higher time intervals (Δt > 6 s), resulting in a concave downward shape.

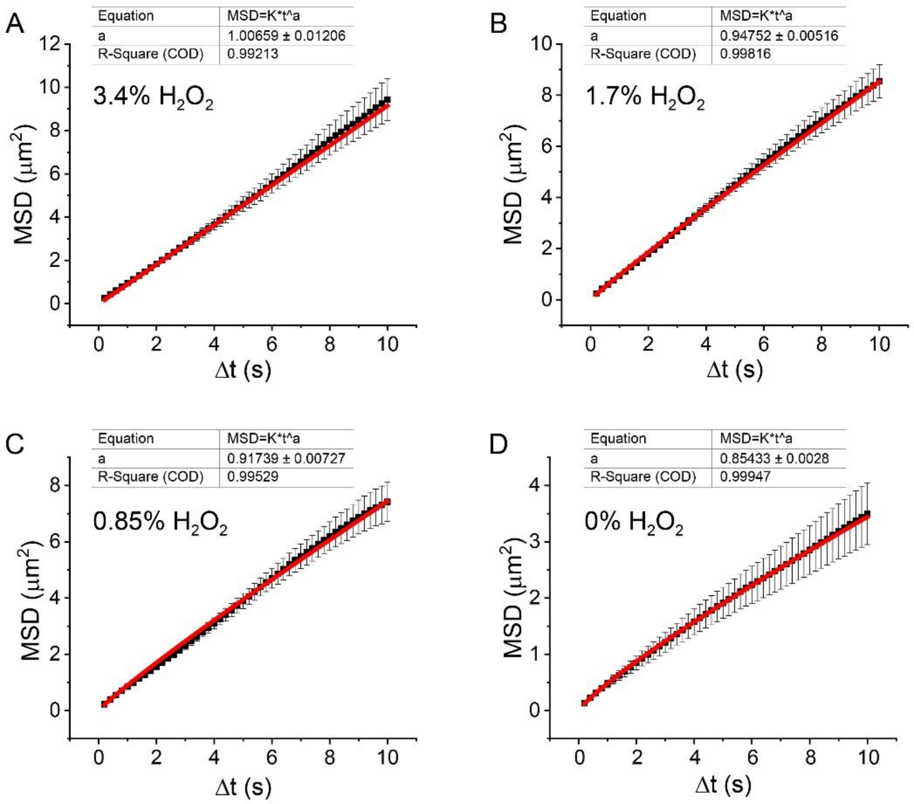

Figure S6. Statistical analysis of anomalous exponent α by fitting MSD curves with equation MSD = $K\Delta t^{\alpha}$. Black curves with error bars represent experimental MSD curves (mean ± SEM, sample number = 40), and red curves represent fitted curves.

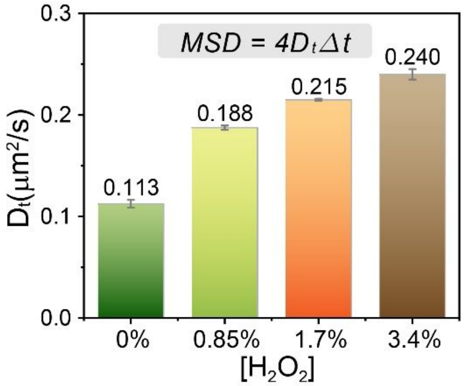

Figure S7. Translational diffusion coefficient values, at different fuel concentrations, extracted by applying the formula MSD = 4Dt∆t to the first 3 seconds of the MSD curves depicted in Figure 3A.

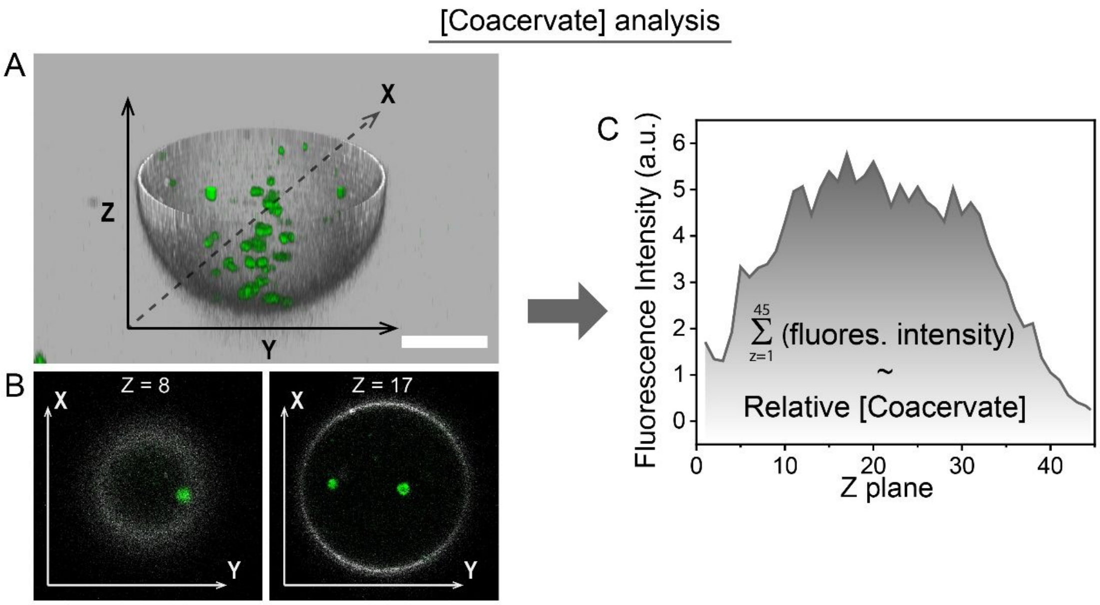

Figure S8. Analysis procedure of the concentration of coacervates in GUVs. A) Reconstructed 3D image of coacervates (green fluorescence) in a GUV (grey fluorescence) from a confocal image stack along the z-axis. B) Representative confocal images used to estimate coacervate concentration with different z-position from one confocal image stack. C) Representative fluorescence intensity across z axis of coacervates in one GUV. Y axis stands for fluorescence intensity of coacervates obtained from one single image out of 45 z images in one confocal image stack. The total fluorescence (sum of 45 fluorescence intensity from 45 z planes) of each GUV (N = 40) was used as an estimation of their relative coacervate concentration, correlated to relative [Coacervate].

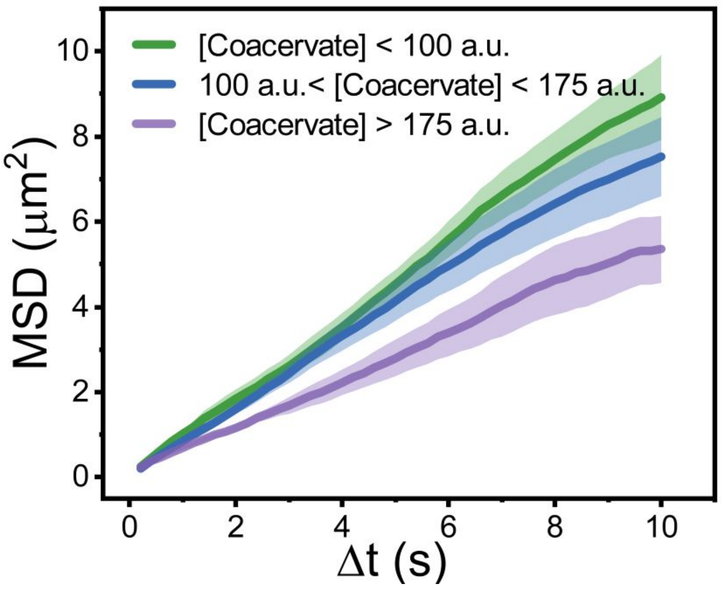

Figure S9. Motility profile of coacervate motors in GUVs at different coacervate concentrations in presence of 0.85% hydrogen peroxide, corresponding to experiments and data shown in Figure 5. The shape of the curve demonstrates (near) diffusive behavior of such confined coacervates regardless of their concentration.

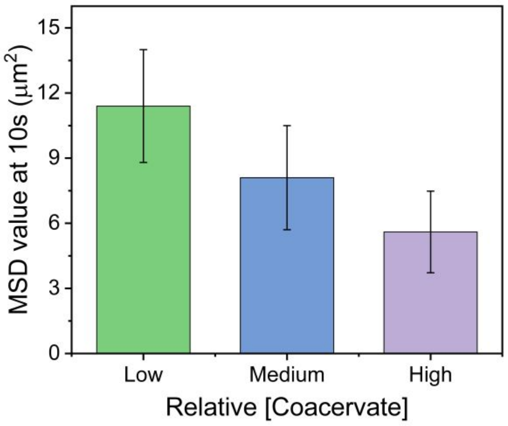

Figure S10. Analysis of MSD values at 10 s for coacervate motors in GUVs of 25-34 μm diameter with 0.85% hydrogen peroxide, for the three [Coacervate] groups: low, medium and high.

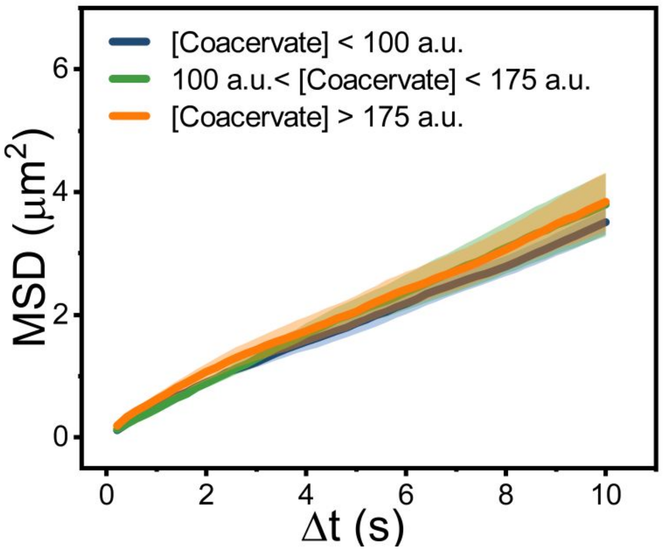

Figure S11. Motility profile of coacervate motors in GUVs at different coacervate concentrations without hydrogen peroxide. No significant differences between the three coacervate concentration groups were observed.

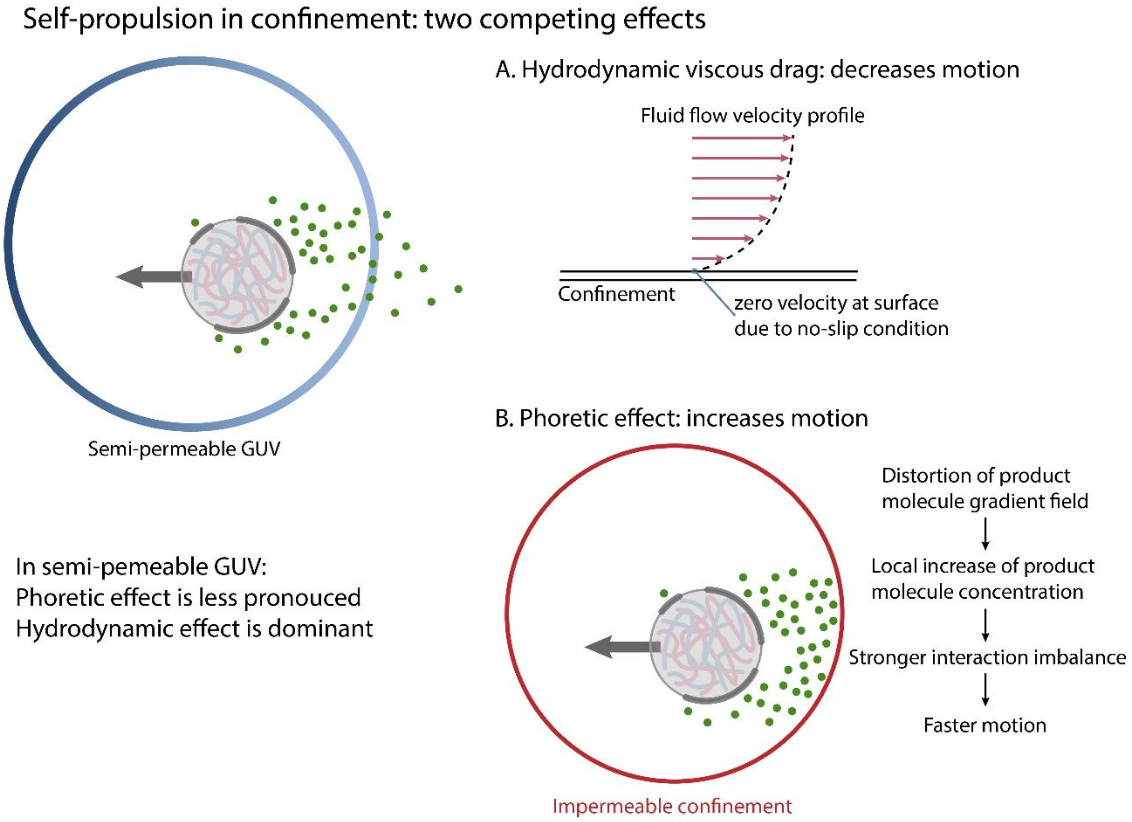

Figure S12. Self-propulsion of self-diffusiophoretic coacervates in GUV confinement. A) No-slip condition of fluid at the confining boundary. The slowdown of fluid is transferred to active coacervates suspended in the fluid, leading to a decrease in motion. B) Impermeable confinement distorts the product molecule gradient field and increases local product concentration, leading to an increase in motion. However, semi-permeable GUVs alleviate the distortion of the product molecule gradient field, resulting in the decelerating hydrodynamic effect being the dominant force.

Movie S1 (separate file). Restrained motion of coacervates in GUVs in the absence of fuel.

Movie S2 (separate file). Self-propulsion of coacervates in GUVs in the presence of fuel. [H_2O_2] = 3.4%

SI References

1. Song, S.; Mason, A. F.; Post, R. A. J.; De Corato, M.; Mestre, R.; Yewdall, N. A.; Cao, S.; van der Hofstad, R. W.; Sanchez, S.; Abdelmohsen, L. K. E. A.; van Hest, J. C. M., Engineering transient dynamics of artificial cells by stochastic distribution of enzymes. *Nat. Commun.* **2021,** *12* (1), 6897.
2. Buddingh', B. C.; Elzinga, K.; van Hest, J. C. M. Intercellular communication between artificial cells by allosteric amplification of a molecular signal. *Nat. Commun.* **2020**, *11*, 1652.
3. Mestre, R (**2021**). Python-based Nano-micromotor Analysis Tool (NMAT) v.1.
4. Mestre, R.; Palacios, L. S.; Miguel-López, A.; Arqué, X.; Pagonabarraga, I.; Sánchez, S. Extraction of the propulsive speed of catalytic nano-and micro-motors under different motion dynamics. *arXiv:2007.15316* **2020**.
